# Neuroradiological findings in Alagille syndrome

**DOI:** 10.1259/bjr.20201241

**Published:** 2021-10-05

**Authors:** Alessandra D'Amico, Teresa Perillo, Renato Cuocolo, Lorenzo Ugga, Fabiola Di Dato, Ferdinando Caranci, Raffaele Iorio

**Affiliations:** 1 Department of Radiology, “Tortorella” Private Hospital, Salerno, Italy; 2 Department of Advanced Biomedical Sciences, University of Naples “Federico II”, Naples, Italy; 3 Department of Clinical Medicine and Surgery, University of Naples "Federico II", Naples, Italy; 4 Department of Translational Medical Science, Section of Pediatrics, University of Naples "Federico II", Naples, Italy; 5 Department of Precision Medicine, University of Campania Luigi Vanvitelli, Naples, Italy

## Abstract

Alagille syndrome (ALGS) is a multisystemic disease caused by mutations in genes of *Notch* pathway, which regulates embryonic cell differentiation and angiogenesis. Clinically, ALGS is characterized by cholestasis, cardiac defects, characteristic facial features, skeletal and ophthalmologic abnormalities. The aim of this review is to illustrate neuroradiological findings in ALGS, which are less well-known and prevalent, including cerebrovascular anomalies (such as aneurysms, dolichoectasia, Moyamoya syndrome and venous peculiarities), Chiari 1 malformation, craniosynostosis, intracranial hypertension, and vertebral anomalies (namely butterfly vertebra, hemivertebra, and craniocervical junction anomalies). Rarer cerebral midline malformations and temporal bone anomalies have also been described.

## Introduction

**Figure 1.**
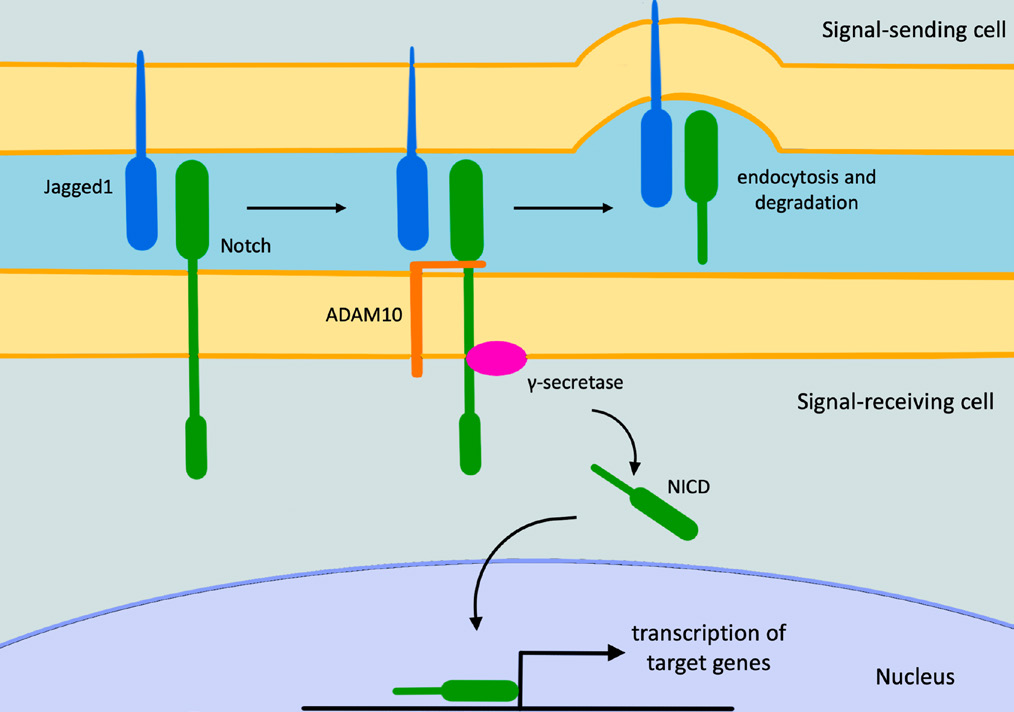
The *Notch* signaling pathway. The pathway starts with *Notch/Jagged1* interaction, causing the *Adam10* metalloprotease activation. This last cleaves *Notch* allowing the *Notch-Jagged1* complex to be endocytosed by the ligand-expressing cell. Subsequently, the γ-secretase enzyme cleaves the remaining part of *Notch* releasing its intracellular domain that moves to the nucleus, where it regulates gene expression. NICD, intracellular domain of the *Notch* protein.

Alagille syndrome (ALGS, OMIM 118450), also known as arteriohepatic dysplasia, is a multisystem autosomal dominant disorder with heterogenous clinical manifestations. The organs mainly affected are liver, heart, skeleton, eyes and kidneys, though endocrine and central nervous system may also be involved. Furthermore, patients usually have characteristic facial features. ALGS has an estimated prevalence of 1:30,000–1:50,000 live births, probably underestimated due to its wide phenotypical variability.^1,2^ Most cases (97%) are caused by heterozygous mutations in *Jaged1 (Jag1*) gene, a cell surface protein that functions as ligand for *Notch* receptors, which plays a crucial role in embryonic cell differentiation and angiogenesis (Figure 1).^3^

ALGS clinical spectrum is wide. The most characteristic pathological and clinical manifestations are cholestasis due to bile duct paucity (interlobular bile ducts are particularly involved), cardiovascular defects, ophthalmologic, skeletal (especially vertebral), and facial abnormalities.

ALGS should be suspected in individuals with at least three of the following five major clinical features: cholestasis, cardiac defects (most commonly stenosis of the peripheral pulmonary artery and its branches), skeletal abnormalities (most commonly butterfly vertebrae), ophthalmologic abnormalities (most commonly posterior embryotoxon), and characteristic facial features (triangular-shaped face with a broad forehead and a pointed chin, bulbous tip of the nose, deeply set eyes, and hypertelorism). Furthermore, bile duct paucity on liver biopsy should be present. To confirm the diagnosis, genetic tests may be required.^4^

Various cerebrovascular anomalies have been associated with ALGS (Table 1). Their knowledge could be useful for the diagnostic work-up of the syndrome even though they are not part of the major diagnostic criteria and are less frequently described in the scientific literature. In this review, we aim to depict all the neuroradiological anomalies reported in ALGS so far.

**Table 1.** Summary table listing facial and craniospinal abnormalities found in patients with ALGS sort by system

System	Abnormalities
Cerebrovascular	Arteries: aneurysms, dolichoectasia, stenosis, Moyamoya syndrome, agenesis of internal carotid artery. Veins: persistent falcine sinus, cerebral veins tortuosity.
Skull	Craniosynostosis Clivus hypoplasia Foveola pharyngica Temporal bone malformations (agenesia/hypoplasia of the posterior semicircular canals, cochlear hypoplasia)
Face	Prominent forehead Deep-set eyes Hypertelorism Straight nose with a flattened tip Large ears Midface hypoplasia Prominent and pointed chin Micrognathia Cleft palate
Brain	Chiari 1 malformation Idiopathic intracranial hypertension Midline malformations (corpus callosum thinning, hypoplasia of the splenium and the isthmus of the corpus callosum, isolated agenesis of the septum pellucidum, bilateral incomplete hippocampal inversion)
Spine	Vertebral anomalies (*e.g.* butterfly vertebra, hemivertebra, segmentation abnormalities) Craniocervical junction abnormalities

ALGS, alagille syndrome.

### Cerebrovascular anomalies

Intracranial arterial and venous anomalies represent a well-recognized feature of ALGS, with an estimated total prevalence of 30–40%.^5^ In particular, arterial abnormalities may cause increased morbidity and mortality, with strokes and intracranial hemorrhage occurring in almost 14% and 14–16% of ALGS patients, respectively.^6,7^

**Figure 2.**
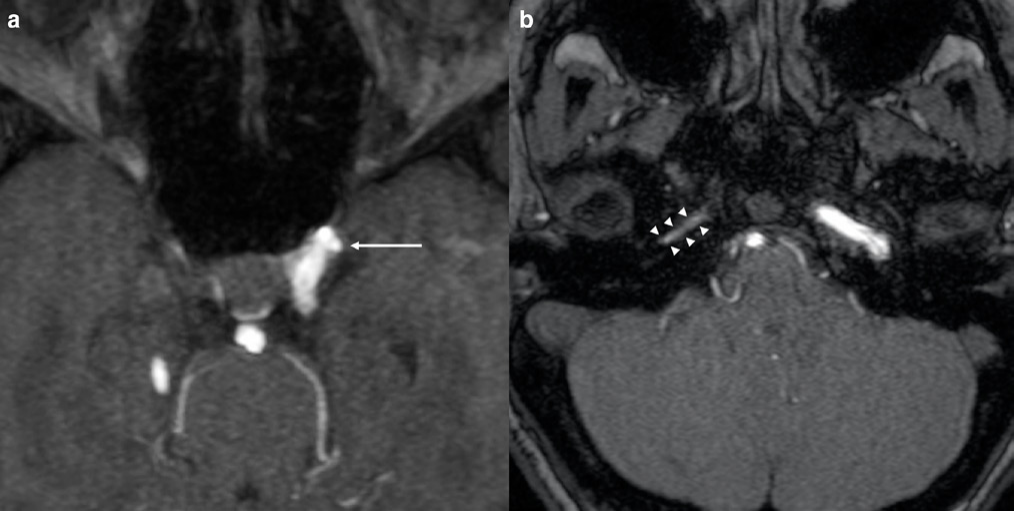
Cerebrovascular anomalies in a 9-year-old girl affected by ALGS. Axial 3D-time of flight MRA of the circle of Willis (a, b) depicts a small aneurysm of the cavernous segment of the left internal carotid artery (*arrow* in a) and narrowing of the petrous segment of the right internal carotid artery (*arrowheads* in b). ALGS, alagille syndrome; MRA, MR angiography.

**Figure 3.**
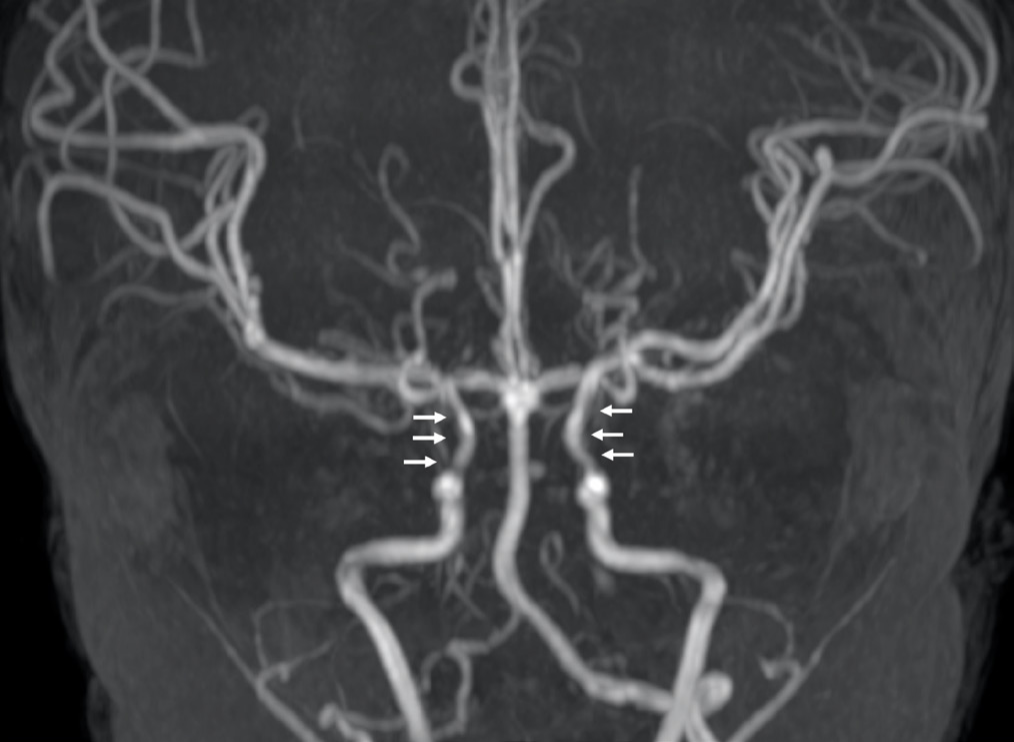
Cerebral arterial anomalies in a 2-year-old boy with ALGS. Coronal 3D-time of flight MRA of the circle of Willis shows stenotic narrowing of the carotid siphons (*arrows*). ALGS, alagille syndrome; MRA, MR angiography.

*Notch* plays an important role in angiogenesis as it is expressed by vascular endothelium during embryogenesis.^8^ This could explain the predisposition to develop vascular abnormalities in ALGS. Intracranial aneurysms have been reported in patients with ALGS in the scientific literature (Figure 2).^7^ This association should always be taken into account due to the risk of aneurysmal rupture and cerebral bleeding.^9^ For instance, Doberentz et al^7^ reported a case of massive and fatal subarachnoid hemorrhage due to the rupture of a large aneurysm of the basilar artery. In addition, cerebral arteries in ALGS have been reported to be more frequently enlarged and elongated or tortuous than in general population. This condition is referred to as dolichoectasia and it has also been reported to occur more frequently in other genetic conditions such as connective tissue disorders (*e.g.* Marfan syndrome, Ehlers-Danlos syndrome) and PHACE syndrome.^10,11^ On the other hand, arterial stenosis (Figure 3) and Moyamoya syndrome (MMS) have been described in ALGS by different authors.^12–15^ MMS is an intracranial arteriopathy causing progressive narrowing and occlusion of the distal part of the internal carotid arteries and their main branches. The compensatory dilation of smaller branches at the base of the brain and in basal ganglia is responsible of the characteristic angiographic appearance called “hazy puff of smoke”, which gives the name to this syndrome.^16^ MMS may cause ischemic stroke in children and hemorrhage in adults.^17^ Another very rare condition described in ALGS is internal carotid agenesis.^18,19^ Kamath et al^20^ evaluated systemic vascular anomalies in 268 ALGS patients. Regarding the cerebrovascular system, they discovered two aneurysms of the basilar artery, one aneurysm of the left middle cerebral artery, one MMS and seven anomalies of the internal carotid arteries (four stenosis, two agenesis and one dolichoectasia). Intracranial bleeds were seen in 14% of patients and accounted for 25% of mortality in their cohort.

**Figure 4.**
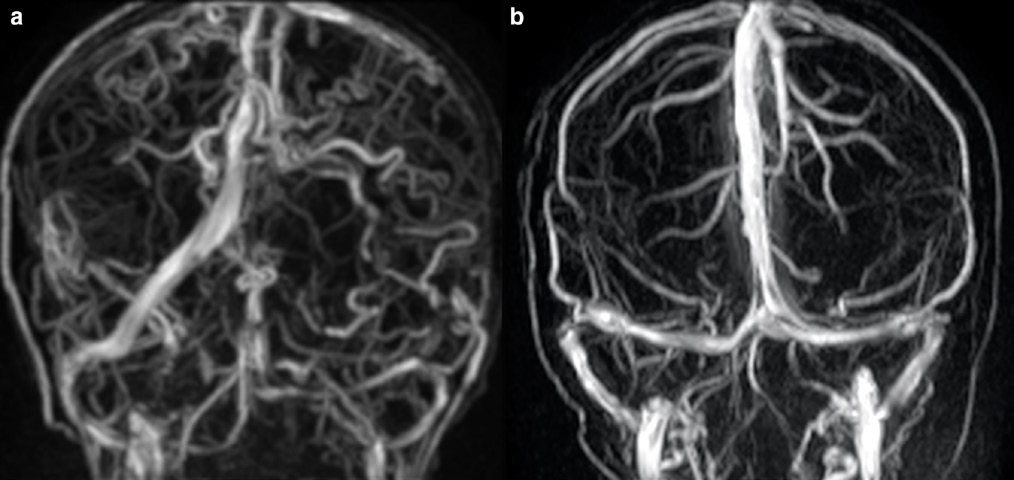
Cerebral venous anomalies in a 15-year-old boy with ALGS. Coronal 3D-phase contrast MRA shows diffuse tortuosity of cerebral veins compared to a child of comparable age without ALGS (b). ALGS, alagille syndrome; MRA, MR angiography.

Carpenter et al^5^ evaluated 19 out of 52 patients with ALGS using different cerebrovascular imaging techniques (MR angiography (MRA), computed tomographic angiography, and digital subtraction angiography) and identified both arterial and venous anomalies. They reported four patients with single or multiple arterial dolichoectasia, three of which also had asymptomatic saccular aneurysms. MMS was discovered in two patients, with one suffering from multiple strokes and the other one from recurrent episodes of bilateral paresthesia and vision blackouts. Regarding venous anomalies, they reported one patient with isolated developmental venous anomaly and three with persistent falcine sinus (the latter with an estimated prevalence of 5.8% compared to 1–2.1% in the general pediatric and adult population). Persistent falcine sinus consists of a normal fetal vein which arises from the straight sinus and drains into the superior sagittal sinus. It may persist in adult age, possibly influencing neurosurgery, in particular in case of transtentorial approach. We report an anomalous case of tortuosity of multiple cerebral veins in a patient affected by ALGS (Figure 4).

Given the genetic predisposition of ALGS patients to cerebrovascular abnormalities, an accurate clinical and neuroradiological screening with brain MRI and MRA of the circle of Willis is recommended as some of these conditions (such as arterial stenoses and aneurysms) are associated with high morbidity and mortality also in absence of prodromal symptoms.^20^

### Skull, facial, and brain anomalies and malformations

Skull malformations represent the second most common bone anomaly in ALGS.^21^ Their prevalence is 0.9%, compared to that of 0.03% occurring in the general population.^22^ Premature fusion of cranial sutures is defined as craniosynostosis and can be simple or complex (if one or more sutures are affected, respectively). Craniosynostosis in ALGS has been reported more frequently as simple, with premature coronal closure being the most common.^23^ A molecular explanation of craniosynostosis in ALGS has been given by Yen et al who demonstrated that *Jag1* plays a key role in skull suture development and fusion.^24^

**Figure 5.**
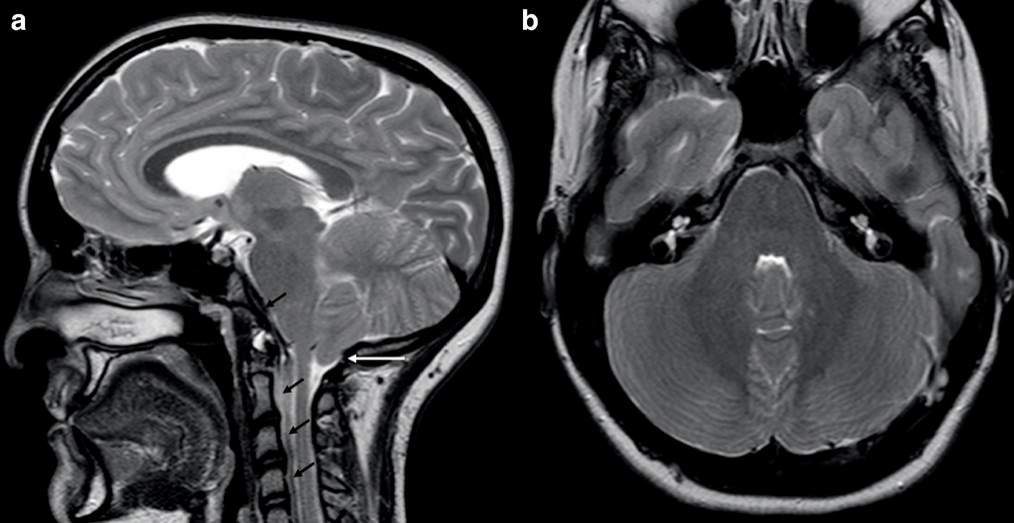
Chiari 1 in a 17-year-old boy with ALGS. Sagittal (a) and axial (b) turbo spin echo *T*_2_ weighted images depicting low lying cerebellar tonsils (white *arrow* in A) and reduced volume of the posterior fossa (b). Clivus and cervical vertebral bodies are hypoplastic (black *arrows* in A). Note also scaphocephaly and frontal bossing. ALGS, alagille syndrome

Chiari 1 has been frequently reported in patients with ALGS, with an estimated prevalence of 33% (Figure 5).^25–27^ This malformation is defined by cerebellar tonsil caudal tip descent of more than 5 mm past the foramen magnum,^28^ whose pathophysiology is still unknown, though it might be partially related to underdevelopment of the bony components of posterior fossa.^29,30^

**Figure 6.**
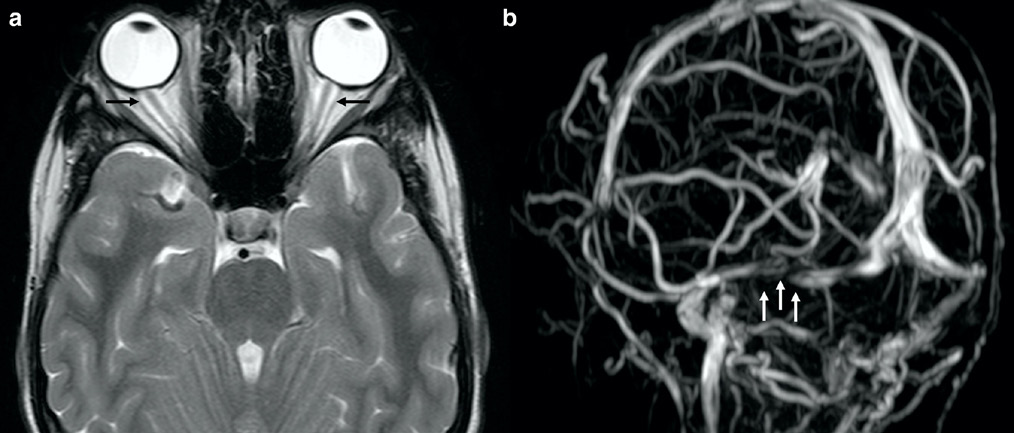
IIH in a 17-year-old boy with ALGS suffering from chronic headache. Axial turbo spin echo *T*_2_ weighted image (a) and coronal 3D-phase contrast MRA (b) depict dilation of the optic nerve sheaths (black *arrows* in A) and a signal gap in the right transverse sinus (white *arrows* in b) due to stenosis, respectively. Also note flattening of the posterior globes contour (a). ALGS, alagille syndrome; IIH, idiopathic intracranial hypertension; MRA, MR angiography.

Idiopathic intracranial hypertension (IIH) has also been reported in ALGS (Figure 6).^31^ Narula et al evaluated 55 ALGS patients, three of which had IIH.^32,33^ Pathogenesis of IIH in ALGS is still not defined but it can be related to craniosynostosis. Furthermore, *Jag1* may play a role in IIH as well, as it may also influence cerebrospinal fluid production due to its involvement in angiogenesis.^34^

Temporal bone malformations have also been reported. Okuno et al^35^ performed a histopathological evaluation of six temporal bones from four pediatric patients with ALGS. All had partial or total absence of the posterior semicircular canals, whereas three had partial absence of the superior ones. The lateral semicircular canals were not affected. In one subject, cochlea was bilaterally shortened. Koch et al^36^ also reported the absence of posterior semicircular canals and hypoplasia of anterior semicircular canals in a patient with ALGS. Although isolated absence of the posterior semicircular canals is overall extremely rare, it could still be a feature of the syndrome. Various genetic syndromes have been frequently associated with semicircular canals anomalies (such as Goldenhaar and CHARGE), whereas posterior semicircular canals aplasia is reported as a characteristic finding of Waardenburg syndrome.^37–39^ Furthermore, Kierman et al^40^ reported that mice with missense mutation of *Jag1* have dysplastic posterior semicircular canals without involvement of the lateral ones.

**Figure 7.**
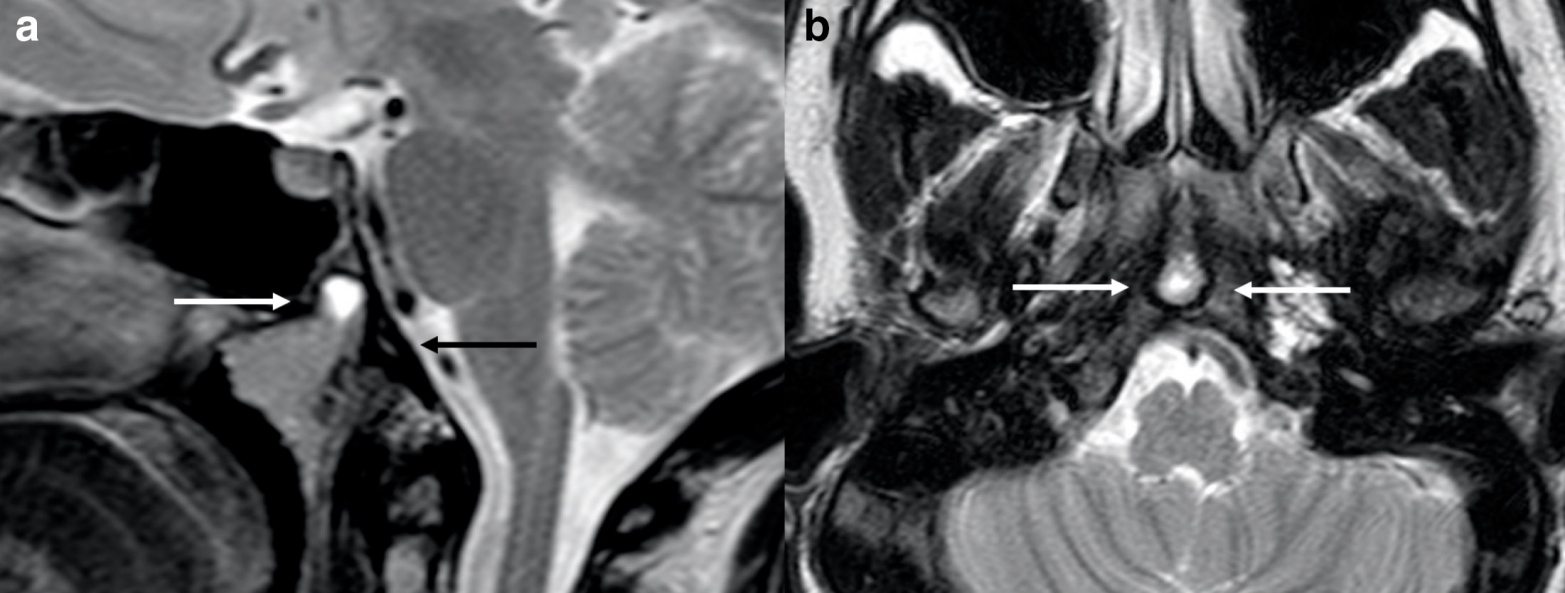
Basicranium anomalies in a 14-year-old girl with ALGS. Sagittal (a) and axial (b) turbo spin echo *T*_2_ weighted images show foveola pharyngica recess (*white arrows* in A and B) and clival hypoplasia (*black arrow* in a). ALGS, alagille syndrome

Regarding skull bone anomalies, here we report one patient with clivus hypoplasia and foveola pharyngica (Figure 7), the latter consisting of persistence of notochordal remnants. It is a rare finding, thought to be a variant of the residual canalis basilaris medianus, which is a round recess in the basiocciput.^41^

Facial abnormalities in ALGS are characteristic and consist of prominent forehead, deep-set eyes, hypertelorism, straight nose with a flattened tip, large ears, midface hypoplasia and prominent and pointed chin.^42,43^ These findings give the overall appearance of the face as an inverted triangle and are often associated with decreased mandibular ramus length and a wide gonion.^44^ Other frequent characteristics are micrognathia and cleft palate.^45^

**Figure 8.**
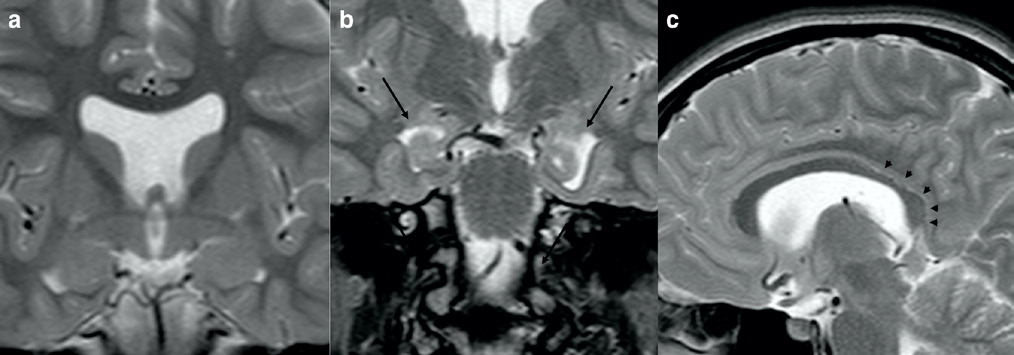
Cerebral midline anomalies in a 15-year-old-girl with ALGS. Coronal turbo spin echo *T*_2_ weighted image shows isolated agenesis of the septum pellucidum (a). Cerebral midline and hippocampal anomalies in a 14-year-old girl with ALGS. Coronal (b) and sagittal (c) turbo spin echo *T*_2_ weighted images demonstrate bilateral incomplete hippocampal inversion (black *arrows* in b) and mild hypoplasia of the splenium and the isthmus of the corpus callosum (black *arrowheads* in c). ALGS, alagille syndrome

Finally, midline cerebral malformations could also be associated with ALGS. Corpus callosum thinning has been reported in rare cases.^27^ A possible genetic explanation is given by the role of *Notch* in corpus callosum development.^46^ We found two cases of midline brain abnormalities, one with isolated agenesis of the septum pellucidum, and one with mild hypoplasia of the splenium and the isthmus of the corpus callosum and concomitant bilateral incomplete hippocampal inversion (Figure 8).

### Spine malformations

**Figure 9.**
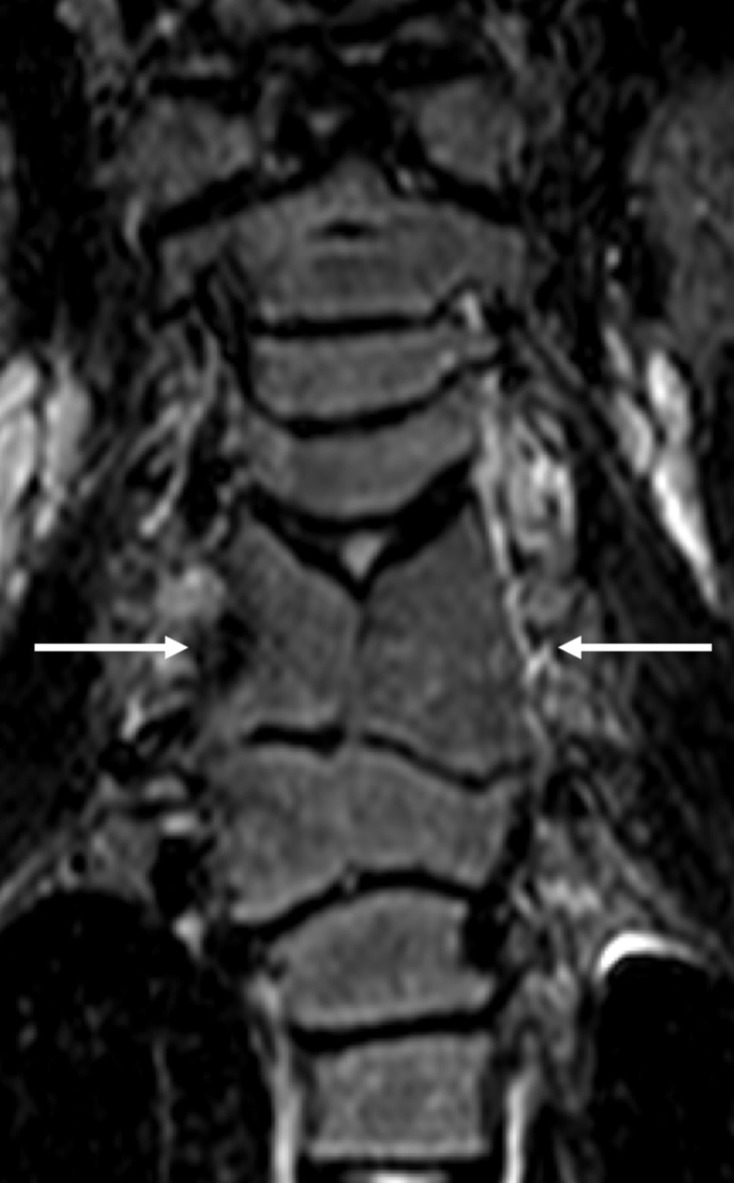
Spine malformations in a 35-year-old male with ALGS. Coronal turbo spin echo *T*_2_ weighted image depicts severe vertebral dysmorphism, consisting of C5 and C6 fusion (segmentation anomaly) and typical median split of the vertebral body (butterfly vertebra) (white *arrows*). ALGS, alagille syndrome

Vertebral anomalies are the most frequent skeletal malformation in ALGS (prevalence of 51–66%), being multiple in 48% of patients.^47,48^ A possible explanation is given by the role played by *Notch* signaling during somitogenesis and craniovertebral junction development.^48–51^ The most frequent vertebral malformations reported in ALGS patients are butterfly vertebra and hemivertebra. Butterfly vertebra is characterized by the presence of a central sagittal cleft in the vertebral body, caused by failed fusion of lateral chondrification centers during spinal embryogenesis due to aberrant persistence of notochordal tissue (Figure 9).^21,52^ It can be complete or partial, depending on presence of a bone bridge across the defect. This malformation is frequently asymptomatic and most commonly affects thoracolumbar spine.^47,48^ Spina bifida occulta has been described only in three patients with ALGS so far.^6,53,54^

**Figure 10.**
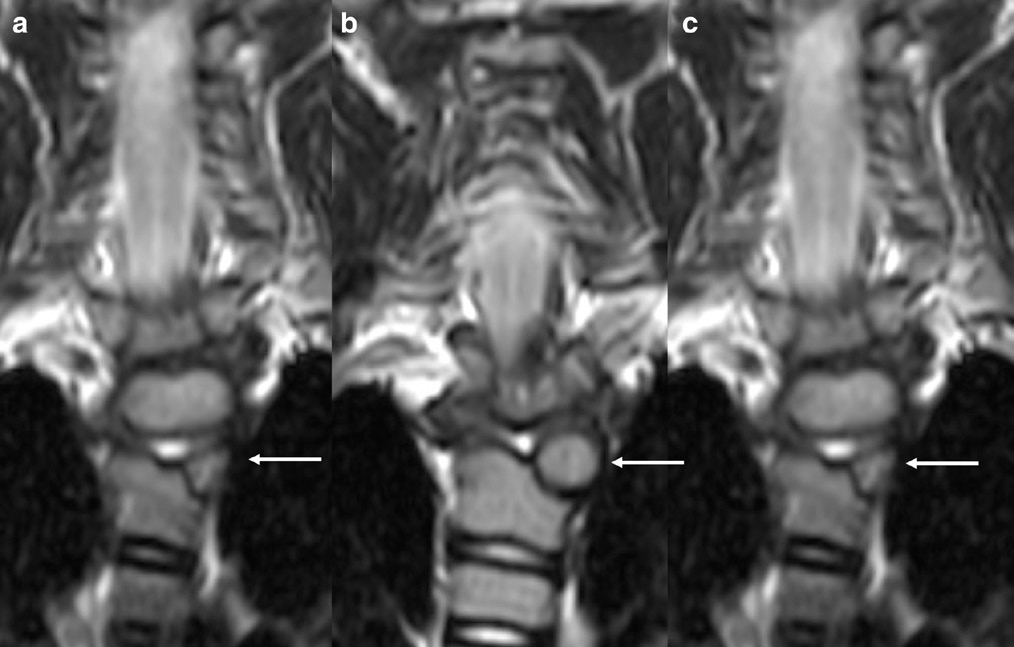
Spine malformation in a 10-year-old boy with ALGS. Coronal turbo spin echo *T*_2_ weighted images (**a–**c) depict a left thoracic hemivertebra (*arrows* in a–c). ALGS, alagille syndrome

Hemivertebra represents agenesis of half of the vertebral body (Figure 10).^55^ It is thought to be caused by lack of development of one of the paired chondrification centers during embryogenesis. Again, thoracic and lumbar spine are more frequently affected, causing kyphosis and/or scoliosis.^56^

Other types of vertebral malformations (such as segmentation anomalies, including block vertebra) have been reported in ALGS as well as anomalies of the craniocervical junction (including partial atlantooccipital joint assimilation).^48,57^

## Conclusions

Craniospinal manifestations of ALGS are extremely wide and neuroradiologists should be familiar with them, especially as some may significantly influence prognosis. In particular, acute complications of vascular stenosis and rupture of aneurysms can cause ischemic strokes and hemorrages, respectively. Therefore, arterial screening with unenhanced brain MRA is suggested in patients with ALGS.

On the other hand, knowledge of the aforementioned neuroradiological findings can further corroborate clinical suspicion of ALGS, thus improving diagnostic work-up.

## References

[1] Danks DM, Campbell PE, Jack I, Rogers J, Smith AL. Studies of the aetiology of neonatal hepatitis and biliary atresia. Arch Dis Child 1977; 52: 360–7. doi: 10.1136/adc.52.5.360 559475 PMC1544552

[2] Saleh M, Kamath BM, Chitayat D. Alagille syndrome: clinical perspectives. Appl Clin Genet 2016; 9: 75–82. doi: 10.2147/TACG.S86420 27418850 PMC4935120

[3] D'Souza B, Miyamoto A, Weinmaster G. The many facets of Notch ligands. Oncogene 2008; 27: 5148–67. doi: 10.1038/onc.2008.229 18758484 PMC2791526

[4] Mitchell E, Gilbert M, Loomes KM. Alagille syndrome. Clin Liver Dis 2018; 22: 625–41. doi: 10.1016/j.cld.2018.06.001 30266153

[5] Carpenter CD, Linscott LL, Leach JL, Vadivelu S, Abruzzo T. Spectrum of cerebral arterial and venous abnormalities in Alagille syndrome. Pediatr Radiol 2018; 48: 602–8. doi: 10.1007/s00247-017-4043-2 29362841

[6] Emerick KM, Rand EB, Goldmuntz E, Krantz ID, Spinner NB, Piccoli DA. Features of Alagille syndrome in 92 patients: frequency and relation to prognosis. Hepatology 1999; 29: 822–9. doi: 10.1002/hep.510290331 10051485

[7] Doberentz E, Kuchelmeister K, Madea B. Subarachnoid hemorrhage due to aneurysm rupture in a young woman with Alagille syndrome – a rare cause of sudden death. Leg Med 2015; 17: 309–12. doi: 10.1016/j.legalmed.2015.03.004 25813756

[8] Leimeister C, Schumacher N, Steidl C, Gessler M. Analysis of HEYL expression in wild-type and Notch pathway mutant mouse embryos. Mech Dev 2000; 98(1–2): 175–8. doi: 10.1016/S0925-4773(00)00459-7 11044625

[9] O'Connell D, Kaliaperumal C, Fanning N, Wyse G, Kaar G. Superior cerebellar aneurysm causing subarachnoid haemorrhage in a 17-year-old with Alagille syndrome. Br J Neurosurg 2012; 26: 287–9. doi: 10.3109/02688697.2011.614022 22026469

[10] Morris SA, Orbach DB, Geva T, Singh MN, Gauvreau K, Lacro RV. Increased vertebral artery tortuosity index is associated with adverse outcomes in children and young adults with connective tissue disorders. Circulation 2011; 124: 388–96. doi: 10.1161/CIRCULATIONAHA.110.990549 21730308

[11] Jia ZY, Zhao LB, Lee DH. Localized marked elongation of the distal internal carotid artery with or without PHACE syndrome: segmental dolichoectasia of the distal internal carotid artery. AJNR Am J Neuroradiol 2018; 39: 817–23. doi: 10.3174/ajnr.A5573 29545249 PMC7410667

[12] Woolfenden AR, Albers GW, Steinberg GK, Hahn JS, Johnston DC, Farrell K. Moyamoya syndrome in children with Alagille syndrome: additional evidence of a vasculopathy. Pediatrics 1999; 103: 505–8. doi: 10.1542/peds.103.2.505 9925853

[13] Connor SEJ, Hewes D, Ball C, Jarosz JM. Alagille syndrome associated with angiographic moyamoya. Childs Nerv Syst 2002; 18(3–4): 186–90. doi: 10.1007/s00381-001-0518-3 11981633

[14] Rocha R, Soro I, Leitão A, Silva ML, Leão M. Moyamoya vascular pattern in Alagille syndrome. Pediatr Neurol 2012; 47: 125–8. doi: 10.1016/j.pediatrneurol.2012.04.014 22759690

[15] Delaney S, O'Connor G, Reardon W, Murphy SJX, Tierney S, Ryan BM, et al. Extracranial and Intracranial Vasculopathy With "Moyamoya Phenomenon" in Association With Alagille Syndrome. Front Neurol 2018; 9: 1194. doi: 10.3389/fneur.2018.01194 30761079 PMC6362309

[16] Ueki K, Meyer FB, Mellinger JF. Moyamoya disease: the disorder and surgical treatment. Mayo Clin Proc 1994; 69: 749–57. doi: 10.1016/S0025-6196(12)61094-5 8035631

[17] Tumialán LM, Dhall SS, Tomak PR, Barrow DL. Alagille syndrome and aneurysmal subarachnoid hemorrhage. Pediatr Neurosurg 2006; 42: 57–61 Available from. doi: 10.1159/000089512 16357504

[18] Kaya O, Yilmaz C, Gulek B, Soker G, Cikman G, Inan I, et al. An important clue in the sonographic diagnosis of internal carotid artery agenesis: ipsilateral common carotid artery hypoplasia. Case Rep Radiol 2014; 2014: 1–4. doi: 10.1155/2014/516456 25097789 PMC4102011

[19] Fitzgerald RT, Zuccoli G. Agenesis of the internal carotid artery: associated malformations including a high rate of aortic and cardiac malformations. Pediatr Radiol 2012; 42: 1333–8. doi: 10.1007/s00247-012-2455-6 22847749

[20] Kamath BM, Spinner NB, Emerick KM, Chudley AE, Booth C, Piccoli DA, et al. Vascular anomalies in Alagille syndrome. Circulation 2004; 109: 1354–8. doi: 10.1161/01.CIR.0000121361.01862.A4 14993126

[21] Berrocal T, Gamo E, Navalo J, Prieto C, Corte P. Pediatric radiology original article syndrome of Alagille: radiological and sonographic findings a review of 37 cases. Eur Radiol 1997; 118: 115–8. 10.1007/s003300050122 9000411

[22] Kamath BM, Stolle C, Bason L, Colliton RP, Piccoli DA, Spinner NB, et al. Craniosynostosis in Alagille syndrome. Am J Med Genet 2002; 112: 176–80 Available from. doi: 10.1002/ajmg.10608 12244552

[23] Narro-Donate JM, Méndez-Román P, Huete-Allut A, Escribano-Mesa JA, Gomar-Alba M, Contreras-Jiménez A, et al. Anterior unilateral plagiocephaly in patient with Alagille syndrome: case report. World Neurosurg 2018; 114: 37–42. doi: 10.1016/j.wneu.2018.03.027 29530693

[24] Yen H-Y, Ting M-C, Maxson RE. Jagged1 functions downstream of Twist1 in the specification of the coronal suture and the formation of a boundary between osteogenic and non-osteogenic cells. Dev Biol 2010; 347: 258–70. doi: 10.1016/j.ydbio.2010.08.010 20727876 PMC3210079

[25] Schwarzenberg SJ, Grothe RM, Sharp HL, Snover DC, Freese D. Long-Term complications of arteriohepatic dysplasia. Am J Med 1992; 93: 171–6. doi: 10.1016/0002-9343(92)90047-F 1497013

[26] Emerick KM, Krantz ID, Kamath BM, Darling C, Burrowes DM, Spinner NB, et al. Intracranial vascular abnormalities in patients with Alagille syndrome. J Pediatr Gastroenterol Nutr 2005; 41: 99–107. doi: 10.1097/01.MPG.0000162776.67758.2F 15990638

[27] Tornincasa C, Di Dato F, D'Amico A, Coppola C, Fedele F, Di Caprio A, et al. P031 neuroradiological abnormalities in a population of asymptomatic children with Alagille syndrome. Dig Liver Dis 2018; 50: e370. doi: 10.1016/S1590-8658(18)31030-2

[28] Hidalgo JA, Tork CA, Varacallo M. Arnold chiari malformation. In: StatPearls [Internet]. Treasure Island (FL): StatPearls Publishing; 2019. https://www.ncbi.nlm.nih.gov/books/NBK431076/. 28613730

[29] Buell TJ, Heiss JD, Oldfield EH. Pathogenesis and cerebrospinal fluid hydrodynamics of the Chiari I malformation. Neurosurg Clin N Am 2015; 26: 495–9. doi: 10.1016/j.nec.2015.06.003 26408057 PMC5140100

[30] Yilmaz S, Turhan T, Mutluer S, Aydogdu S. The association of Alagille syndrome and craniosynostosis. Pediatr Neurol 2013; 48: 146–8. doi: 10.1016/j.pediatrneurol.2012.10.014 23337010

[31] Mouzaki M, Nichter C, Qureshi M, Rountree B, Furuya KN. Idiopathic intracranial hypertension in two patients with Alagille syndrome. J Child Neurol 2010; 25: 1006–8. doi: 10.1177/0883073809351985 20501886

[32] Narula P, Gifford J, Steggall MA, Lloyd C, Van Mourik IDM, Mckiernan PJ, et al. Visual loss and idiopathic intracranial hypertension in children with Alagille syndrome. J Pediatr Gastroenterol Nutr 2006; 43: 348–52. doi: 10.1097/01.mpg.0000221895.51748.44 16954958

[33] Ertekin V, Selimoğlu MA, Tan H. Pseudotumor cerebri due to hypervitaminosis a or hypervitaminosis D or both in Alagille syndrome. Headache 2010; 50: 152–3. doi: 10.1111/j.1526-4610.2009.01489.x 19619235

[34] Krebs LT, Xue Y, Norton CR, Shutter JR, Maguire M, Sundberg JP, et al. Notch signaling is essential for vascular morphogenesis in mice. Genes Dev 2000; 14: 1343–52. 10837027 PMC316662

[35] Okuno T, Takahashi H, Shibahara Y, Hashida Y, Sando I. Temporal bone histopathologic findings in Alagille's syndrome. Arch Otolaryngol Head Neck Surg 1990; 116: 217–20. doi: 10.1001/archotol.1990.01870020093025 2297420

[36] Koch B, Goold A, Egelhoff J, Benton C. Partial absence of the posterior semicircular canal in Alagille syndrome: CT findings. Pediatr Radiol 2006; 36: 977–9. doi: 10.1007/s00247-006-0230-2 16761118

[37] Elmaleh-Bergès M, Baumann C, Noël-Pétroff N, Sekkal A, Couloigner V, Devriendt K, et al. Spectrum of temporal bone abnormalities in patients with Waardenburg syndrome and SOX10 mutations. AJNR Am J Neuroradiol 2013; 34: 1257–63. doi: 10.3174/ajnr.A3367 23237859 PMC7964579

[38] Green GE, Huq FS, Emery SB, Mukherji SK, Martin DM. CHD7 mutations and charge syndrome in semicircular canal dysplasia. Otol Neurotol 2014; 35: 1466–70. doi: 10.1097/MAO.0000000000000260 24979395 PMC4166654

[39] Higashi K, Matsuki C, Sarashina N. Aplasia of posterior semicircular canal in Waardenburg syndrome type II. J Otolaryngol 1992; 21: 262–4. 1527831

[40] Kiernan AE, Ahituv N, Fuchs H, Balling R, Avraham KB, Steel KP, et al. The Notch ligand Jagged1 is required for inner ear sensory development. Proc Natl Acad Sci U S A 2001; 98: 3873–8. doi: 10.1073/pnas.071496998 11259677 PMC31145

[41] Lohman BD, Sarikaya B, McKinney AM, Hadi M. Not the typical Tornwaldt’s cyst this time? A nasopharyngeal cyst associated with canalis basilaris medianus. Br J Radiol 2011; 84: e169–71. doi: 10.1259/bjr/95083086 21849356 PMC3473778

[42] Kamath BM, Loomes KM, Oakey RJ, Emerick KEM, Conversano T, Spinner NB, et al. Facial features in Alagille syndrome: specific or cholestasis facies? Am J Med Genet 2002; 112: 163–70. doi: 10.1002/ajmg.10579 12244550

[43] Humphreys R, Zheng W, Prince LS, Qu X, Brown C, Loomes K, et al. Cranial neural crest ablation of Jagged1 recapitulates the craniofacial phenotype of Alagille syndrome patients. Hum Mol Genet 2012; 21: 1374–83. doi: 10.1093/hmg/ddr575 22156581 PMC3465692

[44] Berniczei-Royko A, Chałas R, Mitura I, Nagy K, Prussak E. Medical and dental management of Alagille syndrome: a review. Med Sci Monit 2014; 20: 476–80. doi: 10.12659/MSM.890577 24658020 PMC3972053

[45] Mackool RL, Shetye P, Grayson B, McCarthy JG. Distraction osteogenesis in a patient with juvenile arthritis. Journal of Craniofacial Surgery 2006; 17: 387–90. doi: 10.1097/00001665-200603000-00034 16633196

[46] Chinn GA, Hirokawa KE, Chuang TM, Urbina C, Patel F, Fong J, et al. Agenesis of the corpus callosum due to defective glial wedge formation in Lhx2 mutant mice. Cereb Cortex 2015; 25: 2707–18. doi: 10.1093/cercor/bhu067 24781987 PMC4537429

[47] Hall M, Borsinger T, Nicholson A, Carter CW. Orthopaedic manifestations of Alagille syndrome: a report of two cases and an updated literature review. JBJS case Connect 2019; 9: e0063. 31688055 10.2106/JBJS.CC.19.00063

[48] Sanderson E, Newman V, Haigh SF, Baker A, Sidhu PS. Vertebral anomalies in children with Alagille syndrome: an analysis of 50 consecutive patients. Pediatr Radiol 2002; 32: 114–9. doi: 10.1007/s00247-001-0599-x 11819079

[49] Shifley ET, Cole SE. The vertebrate segmentation clock and its role in skeletal birth defects. Birth Defects Res C Embryo Today 2007; 81: 121–33. doi: 10.1002/bdrc.20090 17600784

[50] Turnpenny PD, Ellard S. Alagille syndrome: pathogenesis, diagnosis and management. Eur J Hum Genet 2012; 20: 251–7. doi: 10.1038/ejhg.2011.181 21934706 PMC3283172

[51] Pang D, Thompson DNP. Embryology and bony malformations of the craniovertebral junction. Childs Nerv Syst 2011; 27: 523–64. doi: 10.1007/s00381-010-1358-9 21193993 PMC3055990

[52] Kumar R, Guinto FC, Madewell JE, Swischuk LE, David R. The vertebral body: radiographic configurations in various congenital and acquired disorders. Radiographics 1988; 8: 455–85. doi: 10.1148/radiographics.8.3.3380991 3380991

[53] Müller F, O'Rahilly R, Benson DR. The early origin of vertebral anomalies, as illustrated by a 'butterfly vertebra'. J Anat 1986; 149: 157–69. 3693103 PMC1261641

[54] Micaglio E, Andronache AA, Carrera P, Monasky MM, Locati ET, Pirola B, et al. Novel *JAG1* Deletion Variant in Patient with Atypical Alagille Syndrome. Int J Mol Sci 2019; 20: 6247. doi: 10.3390/ijms20246247 31835735 PMC6940840

[55] Johal J, Loukas M, Fisahn C, Chapman JR, Oskouian RJ, Tubbs RS. Hemivertebrae: a comprehensive review of embryology, imaging, classification, and management. Childs Nerv Syst 2016; 32: 2105–9. doi: 10.1007/s00381-016-3195-y 27449768

[56] Wax JR, Watson WJ, Miller RC, Ingardia CJ, Pinette MG, Cartin A, et al. Prenatal sonographic diagnosis of Hemivertebrae. J Ultrasound Med 2008; 27: 1023–7. doi: 10.7863/jum.2008.27.7.1023 18577665

[57] Xu R, Xia Y, Passias PG, Protopsaltis T, Sciubba DM. Occipitocervical osteotomies and Interfacet grafts for reduction of occipitocervical kyphosis and basilar invagination. World Neurosurg 2019; 127: 391–6. doi: 10.1016/j.wneu.2019.03.271 30954738

